# Supplementary material for: Use of the Species Sensitivity Distribution Approach to Derive Ecological Threshold of Toxicological Concern (eco-TTC) for Pesticides
Source: Int J Environ Res Public Health. 2021 Nov 17;18(22):12078. doi: 10.3390/ijerph182212078 (PMC8623465; doi:10.3390/ijerph182212078)
Supplement: Supplementary file 1 [file ijerph-18-12078-s001.zip › ijerph-1432749-supplementary.pdf]

SUPPORTING INFORMATION

Article

Use of the Species Sensitivity Distribution Approach to Derive Ecological Threshold of Toxicological Concern (eco-TTC) for Pesticides

Cristiana Rizzi 1, Sara Villa 1,*, Alessandro Sergio Cuzzeri 1 and Antonio Finizio 1

1 Department of Earth and Environmental Sciences DISAT, University of Milano-Bicocca, Piazza della Scienza 1, 20126 Milano, Italy; cristiana.rizzi@unimib.it; sara.villa@unimib.it; a.cuzzeri@campus.unimib.it; antonio.finizio@unimib.it

* Correspondence: sara.villa@unimib.it

Table S1- Selected chemical physical properties of the investigated insecticides

Chemical class	Substances	M.W.	log Kow	References
carbamates	carbaryl	201.22	2.36	PPDB (Pesticide Properties Database)
	carbofuran	221.25	2.32	Hansch et al., 1995
	carbosulfan	380.5	5.4	WHO/FAO, 2004
	fenobucarb	207.27	2.78	PPDB (Pesticide Properties Database)
neonicotinoids	clothianidin	249.68	0.905	PPDB (Pesticide Properties Database)
	dinotefuran	202.21	-0.549	PPDB (Pesticide Properties Database)
	imidacloprid	255.66	0.57	PPDB (Pesticide Properties Database)
	thiacloprid	252.72	1.26	PPDB (Pesticide Properties Database)
	thiamethoxam	291.71	-0.13	PPDB (Pesticide Properties Database)
cyclodienes and HCHs	chlordane	409.8	6.0	EFSA, 2007
	chlordecone	490.6	4.5	UNEP, 2007
	dieldrin	380.9	5.4	Finizio et al., 1997
	endosulfan	406.9	3.62	Hansch et al., 1995
	endrin	380.9	5.2	Finizio et al., 1997
	heptachlor	373.3	5.4	Verschueren, 2001
	strobane	377.3	4.3	
	toxaphene	414	5.9	Fisk et al., 1999)
	α-HCH	290.83	3.7	Finizio et al., 1997
	γ-HCH	290.83	3.7	Finizio et al., 1997
organophosphorous	azinphos-methyl	317.3	2.96	PPDB (Pesticide Properties Database)

	chlorpyrifos	350.6	4.7	PPDB (Pesticide Properties Database)
	diazinon	304.35	3.69	PPDB (Pesticide Properties Database)
	fenitrothion	277.24	3.3	PPDB (Pesticide Properties Database)
	fenthion	278.3	4.2	Finizio et al., 1997
	malathion	330.4	2.75	PPDB (Pesticide Properties Database)
	parathion-ethyl	291.26	3.83	PPDB (Pesticide Properties Database)
	parathion-methyl	263.21	3	PPDB (Pesticide Properties Database)
	phenthoate	320.4	3.69	PPDB (Pesticide Properties Database)
pyrethroids	bifenthrin	422.9	6	Hansch et al.,1995
	cyfluthrin	434.3	6	PPDB (Pesticide Properties Database)
	cypermethrin	416.3	6.3	Finizio et al., 1997
	deltamethrin	505.2	6.5	Finizio et al., 1997
	etofenprox	376.5	6.9	PPDB (Pesticide Properties Database)
	fenvalerate	419.9	6.2	Hansch et al.,1995
	γ-cyalothrin	449.8	6.8	PPDB (Pesticide Properties Database)
	permethrin	391.3	6.5	Finizio et al., 1997
	λ-cyhalothrin	449.8	6.8	PPDB (Pesticide Properties Database)
miscellaneous	cartap	237.3	0.2	Roberts and Hutson, 1999
	chlorantraniliprole	483.1	2.86	PPDB (Pesticide Properties Database)
	dicofol (acaricide)	370.5	5.74	Han et al., 2011
	diflubenzuron	310.68	3.89	PPDB (Pesticide Properties Database)
	DNOC	198.13	2.39	PPDB (Pesticide Properties Database)
	fipronil	437.1	3.75	PPDB (Pesticide Properties Database)
	methoxychlor	345.6	5.83	Mnif et al., 2011
	p,p' - DDT	354.49	6.91	Finizio et al., 1997
	spinosad	1477.9	4.2	Roberts and Hutson, 1999
	tebufenozide	352.5	4.25	PPDB (Pesticide Properties Database)
	thiocyclam	181.4	1.3	Roberts and Hutson, 1999

Table S2- Selected chemical physical properties of the investigated herbicides

**Chemical class**	**Substance**	**M.W.**	**log Kow**	**References**
sulfonylureas	bensulfuron-methyl	410.4	2.45	Gandy et al., 2015
	cyclosulfamuron	421.4	2.05	Gandy et al., 2015
	imazosulfuron	412.8	-0.07	PPDB (Pesticide Properties Database)
	propyrisulfuron	455.9	2.9	PPDB (Pesticide Properties Database)
	pyrazosulfuron-ethyl	414.4	1.3	Gandy et al., 2015
triazines	atrazine	215.68	2.6	Finizio et al., 1997
	irgarol (biocide)	253.37	3.95	O'Neil, 2013
	metamitron	202.21	0.85	PPDB (Pesticide Properties Database)
	metribuzin	214.29	1.9	Finizio et al., 1997
	prometryn	241.36	3.3	Finizio et al., 1997
	simazine	201.657	2.2	Finizio et al., 1997
	simetryn	213.31	2.8	PPDB (Pesticide Properties Database)
	terbuthylazine	229.71	3.4	PPDB (Pesticide Properties Database)
	terbutryn	241.36	3.7	Finizio et al., 1997
miscellaneous	2,4-D	221.03	-0.82	PPDB (Pesticide Properties Database)
	benfuresate	256.32	2.41	PPDB (Pesticide Properties Database)
	bentazone	240.28	2.34	Gandy et al., 2015
	benzofenap	431.3	4.69	PPDB (Pesticide Properties Database)
	butachlor	311.8	4.5	PPDB (Pesticide Properties Database)
	cafenstrole	350.4	3.21	PPDB (Pesticide Properties Database)
	carfentrazone-ethyl	412.2	3.7	PPDB (Pesticide Properties Database)
	diquat	184.24	0.27	Gandy et al., 2015
	esprocarb	265.4	4.6	PPDB (Pesticide Properties Database)
	fentrazamide	349.81	3.6	PPDB (Pesticide Properties Database)
	indanofan	340.8	3.59	PPDB (Pesticide Properties Database)
	mefenacet	298.4	3.23	PPDB (Pesticide Properties Database)
	metolachlor	283.79	3.28	Ellgehausen.et al., 1981
	molinate	187.3	3.2	Finizio et al., 1997
	oxadiargyl	341.2	3.95	PPDB (Pesticide Properties Database)
	pendimethalin	281.31	5.4	PPDB (Pesticide Properties Database)
	pentoxazone	353.8	4.66	PPDB (Pesticide Properties Database)
	pretilachlor	311.8	4.08	PPDB (Pesticide Properties Database)

	pyraclonil	314.77	1.61	PPDB (Pesticide Properties Database)
	pyrazoxyfen	403.3	3.69	PPDB (Pesticide Properties Database)
	pyriminobac-methyl	361.3	2.31	PPDB (Pesticide Properties Database)
	pyrimisulfan	419.4	1.88	Gandy et al., 2015
	quinoclamine	207.61	2.12	Gandy et al., 2015
	tefuryltrione	442.9	-0.05	Matsushita et al., 2018
	thiobencarb	257.779	4.23	PPDB (Pesticide Properties Database)
ureas	daimuron	268.35	2.7	PPDB (Pesticide Properties Database)
	isoproturon	206.3	2.87	Leo, 1998
	linuron	249.091	2.8	Finizio et al., 1997
	diuron	233.1	2.7	Finizio et al., 1997
	cumyluron	302.8	3.71	Gandy et al., 2015

Table S3- Selected chemical physical properties of the investigated fungicides

Chemical class	Substance	M.W.	log Kow	References
azoles	cyproconazole	291.77	2.94	PPDB (Pesticide Properties Database)
	epoxiconazole	329.8	3.3	PPDB (Pesticide Properties Database)
	fenbuconazole	336.8	3.79	PPDB (Pesticide Properties Database)
	hydroxyisoxazole	99.09	0.46	USEPA (CompTox database)
	myclobutanil	288.77	2.89	PPDB (Pesticide Properties Database)
	propiconazole	342.2	3.72	PPDB (Pesticide Properties Database)
	tebuconazole	307.82	3.7	PPDB (Pesticide Properties Database)
	tricyclazole	189.4	1.4	PPDB (Pesticide Properties Database)
carbamates	benomyl	290.32	1.4	PPDB (Pesticide Properties Database)
	carbendazim	191.19	1.48	PPDB (Pesticide Properties Database)
	mancozeb	541.1	2.33	PPDB (Pesticide Properties Database)
	maneb	265.3	-0.45	PPDB (Pesticide Properties Database)
	metiram (Zineb)	275.8	0.33	PPDB (Pesticide Properties Database)
	thiram	240.4	1.84	PPDB (Pesticide Properties Database)
	ziram	305.8	1.65	PPDB (Pesticide Properties Database)
strobilurines	azoxystrobin	403.4	2.5	PPDB (Pesticide Properties Database)
	fluoxastrobin	458.8	2.86	PPDB (Pesticide Properties Database)
	kresoxim-CH_3	313.3	3.4	PPDB (Pesticide Properties Database)
	picoxystrobin	367.3	3.68	PPDB (Pesticide Properties Database)
	trifloxystrobin	408.4	4.5	PPDB (Pesticide Properties Database)
miscellaneous	anilazine	275.5	3.88	PPDB (Pesticide Properties Database)
	captafol	349.1	3.8	Finizio et al., 1997
	captan	300.6	2.5	Finizio et al., 1997
	chlorothalonil	265.9	3.05	PPDB (Pesticide Properties Database)
	cyprodinil	225.29	4	PPDB (Pesticide Properties Database)
	dithianon	296.3	3.2	PPDB (Pesticide Properties Database)
	dodine	287.44	1.25	PPDB (Pesticide Properties Database)
	fluazinam	465.09	4.87	PPDB (Pesticide Properties Database)
	fludioxonil	248.18	4.12	PPDB (Pesticide Properties Database)

	flutolanil	323.31	3.17	PPDB (Pesticide Properties Database)
	folpet	296.6	3.02	PPDB (Pesticide Properties Database)
	hexachlorobutadiene	260.8	4.78	USEPA (CompTox database)
	HCB	284.8	5.73	USEPA (CompTox database)
	iprobenfos	288.34	3.37	PPDB (Pesticide Properties Database)
	isoprothiolane	290.4	3.3	PPDB (Pesticide Properties Database)
	phthalide	134.13	3.01	PPDB (Pesticide Properties Database)
	probenazole	223.25	1.4	PPDB (Pesticide Properties Database)
	pyrazophos	373.37	3.8	PPDB (Pesticide Properties Database)
	pyroquilon	173.21	1.57	PPDB (Pesticide Properties Database)
	tolylfluanid	347.3	3.9	PPDB (Pesticide Properties Database)

Table S4 – Selection criteria utilized when, for the same compound, more than one HC_5 value was found in literature

Chemical class	Chemical	HC_5 ($\mu g\ L^{-1}$)	N° endpoints (SSD)	Toxicological endpoint	Tested organisms	References	Selected value	Selection criteria
benzimidazoles	carbendazim	8	16	EC50	aq. comm.	Maltby et al., 2009	**8**	worst case, larger data set
		9.8	7	LC50	invertebrates, temperate	Rico et al.; 2011		
benzoylureas	diflubenzuron	0.05	16	LC50+EC50	arthropods	Nagai, 2016	**0.05**	worst case, larger data set
		0.05	>6	EC50	arthropods	Maltby et al., 2005		
		23.2	>6	EC50	vertebrates	Maltby et al., 2005		
bromides	diquat	0.6	13	EC50	plants	Lewis and Thursby, 2018	**0.6**	worst case
		3.5	17	EC50	plants	van den Brink, et al., 2006		
carbamates	carbofuran	0.23	>6	EC50	arthropods	Maltby et al., 2005	**0.23**	worst case
		1.6	9	LC50 + EC50	arthropods	Nagai, 2016		
		68	>6	EC50	vertebrates	Maltby et al., 2005		
	carbaryl	2.67	>6	EC50	arthropods	Maltby et al., 2005	**2.67**	worst case
		314	>6	EC50	non-arthr. invertebrates	Maltby et al., 2005		
		450	>6	EC50	vertebrates	Maltby et al., 2005		
chloroacetanilides	metolachlor	30.8	28	EC50	plants	Lewis and Thursby, 2018	**30.8**	worst case, larger data set
		1784	11	EC50	diatoms	Larras et al.; 2012		
chloronitriles	chlorothalonil	2.5	25	LC50 + EC50	aq.comm	Nagai, 2016	**6**	the two availabe values are similar, however, 6 ug/L was obtained using a larger data set
		6	46	EC50	aq.comm	Maltby et al., 2009		
organochlorines	methoxychlor	0.47	>6	EC50	arthropods	Maltby et al., 2005	**1.15**	larger data set
		1.15	85	LC50	aq.comm	Wang et al.; 2008		
		4.6	>6	EC50	vertebrates	Maltby et al., 2005		
organophosphates	malathion	0.45	76	LC50	arthropods, temperate	Rico et al.; 2011	**0.45**	worst case, larger data set
		53	36	LC50	fish, temperate	Rico et al.; 2011		
		57.6	5	LC50	fish, amazon	Rico et al.; 2011		

	chlorpyrifos	0.07	>6	EC50	arthropods	Maltby et al., 2005	**0.034**	worst case
		0.58	>6	EC50	vertebrates	Maltby et al., 2005		
		0.034	23	EC50	crustacea	Giddings et al., 2014		
		0.087	17	EC50	insects	Giddings et al., 2014		
		0.81	25	LC50	vertebrates	Giddings et al., 2014		
	fenitrothion	0.32	>6	EC50	arthropods	Maltby et al., 2005	**0.32**	worst case
		1.4	37	LC50 + EC50	arthropods	Nagai, 2016		
		8.16	>6	EC50	non-arthr. invertebrates	Maltby et al., 2005		
		53	>6	EC50	vertebrates	Maltby et al., 2005		
		790	>6	EC50	plants	Maltby et al., 2005		
	diazinon	0.36	>6	EC50	arthropods	Maltby et al., 2005	**0.36**	worst case
		1.3	23	LC50 + EC50	arthropods	Nagai, 2016		
		52	>6	EC50	vertebrates	Maltby et al., 2005		
		229	>6	EC50	non-arthr. invertebrates	Maltby et al., 2005		
	azinphos-CH_3	0.04	>6	EC50	arthropods	Maltby et al., 2005	**0.04**	worst case
		0.36	>6	EC50	vertebrates	Maltby et al., 2005		
	parathion-C_2H_5	0.23	>6	EC50	arthropods	Maltby et al., 2005	**0.23**	worst case
		176	>6	EC50	non-arthr. invertebrates	Maltby et al., 2005		
		93	>6	EC50	vertebrates	Maltby et al., 2005		
	parathion-CH_3	0.31	>6	EC50	arthropods	Maltby et al., 2005	**0.31**	worst case
		1.5	>6	EC50	vertebrates	Maltby et al., 2005		
		754	>6	EC50	non-arthr. invertebrates	Maltby et al., 2005		
phenylureas	diuron	1.43	11	EC50	diatoms	Larras et al.; 2012	**1.9**	the two availabe values are quite similar, however, 1.9 µg L^{-1} was obtained using a larger data set
		1.9	46	EC50	plants	Larras et al.; 2012		
	linuron	5.8	8	EC50	plants	van den Brink, et al., 2006	**3.4**	

		3.4	10	EC50	plants	Lewis and Thursby, 2018		worst case, larger data set
pyrethroids	λ-cyhalothrin	0.003	>6	EC50	arthropods	Maltby et al., 2005	**0.003**	worst case
		0.08	>6	EC50	vertebrates	Maltby et al., 2005		
	cypermethrin	0.003	>6	EC50	arthropods	Maltby et al., 2005	**0.003**	worst case
		0.17	>6	EC50	vertebrates	Maltby et al., 2005		
	deltamethrin	0.009	>6	EC50	arthropods	Maltby et al., 2005	**0.009**	worst case
		0.21	>6	EC50	vertebrates	Maltby et al., 2005		
	permethrin	0.096	>6	EC50	arthropods	Maltby et al., 2005	**0.096**	worst case
		0.39	>6	EC50	vertebrates	Maltby et al., 2005		
	fenvalerate	0.013	>6	EC50	arthropods	Maltby et al., 2005	**0.013**	worst case
		0.19	>6	EC50	vertebrates	Maltby et al., 2005		
triazines	metribuzin	7.4	19	EC50	plants	van den Brink, et al., 2006	**7.4**	the two availabe values are similar, however, 7.4 µg L^{-1} was obtained using a larger data set
		7.6	18	EC50	plants	Lewis and Thursby, 2018		
	atrazine	13	29	EC50	plants	van den Brink, et al., 2006	**16.2**	the two availabe values are similar, however, 16.2 µg L^{-1} was obtained using a larger data set
		16.2	69	EC50	plants	Lewis and Thursby, 2018		
		203	11	EC50	diatoms	Larras et al.; 2012		

Table S5- Insecticides: selected HC_5 values (expressed both as μg L^{-1} and μmol L^{-1}), number and typologies of tested species to derive the SSD curves

**Chemical class**	**Substance**	**HC_5 (μg L^{-1})**	**HC_5 (μmol L^{-1})**	**Endpoint(s)**	**N° endpoints (SSD)**	**Tested organisms**	**References**
carbamates	carbaryl	2.67	1.33E-02	EC50	>6	arthropods	Maltby et al.; 2005
	carbofuran	0.23	1.04E-03	EC50	>6	arthropods	Maltby et al.; 2005
	carbosulfan	0.32	8.41E-04	EC50 and LC50	8	invertebrates	Nagai, 2016
	fenobucarb	2.9	1.40E-02	EC50 and LC50	22	invertebrates	Nagai, 2016
neonicotinoids	clothianidin	0.56	2.24E-03	EC50 and LC50	6	arthropods	Nagai, 2016
	dinotefuran	3.8	1.88E-02	EC50 and LC50	7	arthropods	Nagai, 2016
	imidacloprid	0.51	1.99E-03	EC50 and LC50	16	arthropods	Nagai, 2016
	thiacloprid	1.6	6.33E-03	EC50 and LC50	8	arthropods	Nagai, 2016
	thiamethoxam	7.1	2.43E-02	EC50 and LC50	7	arthropods	Nagai, 2016
cyclodienes and HCHs	chlordane	4.9	1.20E-02	LC50	57	invert and vert.	Wang et al.; 2008
	chlordecone	18.0	3.66E-02	LC50	24	invert and vert.	Wang et al.; 2008
	dieldrin	1.5	4.05E-03	LC50	105	invert and vert.	Wang et al.; 2008
	endosulfan	0.2	5.64E-04	LC50	124	invert and vert.	Wang et al.; 2008
	endrin	0.1	1.83E-04	LC50	121	invert and vert.	Wang et al.; 2008
	heptachlor	2.2	5.95E-03	LC50	65	invert and vert.	Wang et al.; 2008
	strobane	3.6	9.66E-03	LC50	12	invert and vert.	Wang et al.; 2008
	toxaphene	1.3	3.02E-03	LC50	64	invert and vert.	Wang et al.; 2008
	α-HCH	126	4.3E-01	LC50	18	invert and vert.	Wang et al.; 2008

	γ-HCH	3.7	1.26E-02	LC50	145	invert and vert.	Wang et al.; 2008
organophosphorous	azinphos-methyl	0.04	1.26E-04	EC50	>6	arthropods	Maltby et al.; 2005
	chlorpyrifos	0.034	9.7E-05	EC50	23	crustaceans	Giddings et al., 2014
	diazinon	0.36	1.18E-03	EC50	>6	arthropods	Maltby et al.; 2005
	fenitrothion	0.32	1.15E-03	EC50	>6	arthropods	Maltby et al.; 2005
	fenthion	0.41	1.47E-03	EC50 and LC50	31	arthropods	Nagai, 2016
	malathion	0.45	1.36E-03	LC50	76	arthropods	Rico et al.; 2011
	parathion-ethyl	0.23	7.90E-04	EC50	>6	arthropods	Maltby et al.; 2005
	parathion-methyl	0.31	1.18E-03	EC50	>6	arthropods	Maltby et al.; 2005
	phenthoate	0.22	6.87E-04	EC50 and LC50	11	arthropods	Nagai, 2016
pyrethroids	bifenthrin	0.004	9.46E-06	EC50 and LC50	55	arthropods	UCDAVIS, 2010a
	cyfluthrin	0.002	4.61E-06	EC50 and LC50	40	AQCOM	UCDAVIS, 2010b
	cypermethrin	0.003	7.21E-06	EC50	>6	arthropods	Maltby et al.; 2005
	deltamethrin	0.009	1.78E-05	EC50	>6	arthropods	Maltby et al.; 2005
	etofenprox	0.085	1,54E-04	EC50 and LC50	12	arthropods	Nagai, 2016
	fenvalerate	0.013	3.10E-05	EC50	>6	arthropods	Maltby et al.; 2005
	γ-cyalothrin	0.0029	6.45E-06	EC50 and LC50	8	invertebrates	Van Wijngaarden et al.; 2009
	permethrin	0.096	2.45E-04	EC50	>6	arthropods	Maltby et al.; 2005
	λ-cyhalothrin	0.003	6.67E-06	EC50	>6	arthropods	Maltby et al.; 2005
miscellaneous	cartap	3	1.26E-02	EC50 and LC50	13	arthropods	Nagai, 2016

	chlorantraniliprole	5.5	1.14E-02	EC50 and LC50	11	arthropods	Nagai, 2016
	dicofol (acaricide)	58.1	1.57E-01	LC50	27	invert and vert.	Wang et al.; 2008
	diflubenzuron	0.05	1.48E-04	EC50	16	arthropods	Nagai, 2016
	DNOC	36	1.82E-01	EC50	9	AQCOM*	Maltby et al.; 2009
	fipronil	0.1	2.29E-04	EC50 and LC50	21	arthropods	Nagai, 2016
	methoxychlor	1.2	3.91E-03	LC50	85	invert and vert.	Wang et al.; 2008
	p,p' - DDT	0.9	2.56E-03	LC50	207	invert and vert.	Wang et al.; 2008
	spinosad	0.74	5.01E-04	EC50 and LC50	7	arthropods	Nagai, 2016
	tebufenozide	220	6.24E-01	EC50 and LC50	8	arthropods	Nagai, 2016
	thiocyclam	6.1	3.36E-02	EC50 and LC50	7	arthropods	Nagai, 2016

*AQCOM = Aquatic Community

Table S6 - Herbicides: selected HC_5 values (expressed both as µg L^{-1} and µmol L^{-1}), number and typologies of tested species to derive the SSD curves

Chemical class	Substance	HC_5 (µg L^{-1})	HC_5 (µmol L^{-1})	Endpoint(s)	N° endpoints (SSD)	Tested organisms	References
sulfonylureas	bensulfuron-CH_3	0.35	8.53E-04	EC50 and LC50	11	plants	Nagai, 2016
	cyclosulfamuron	0.28	6.64E-04	EC50 and LC50	6	plants	Nagai, 2016
	imazosulfuron	4.5	1.09E-02	EC50 and LC50	8	plants	Nagai, 2016
	propyrisulfuron	15	3.29E-02	EC50 and LC50	6	plants	Nagai, 2016
triazines	atrazine	16.2	7.51E-02	EC50	69	plants	Lewis and Thursby, 2018
	metribuzin	7.5	3.50E-02	EC50	18	plants	Lewis and Thursby, 2018
	prometryn	1.3	5.39E-03	EC50	13	plants	Lewis and Thursby, 2018
	simazine	52	2.58E-01	EC50	10	plants	van den Brink, et al., 2006
	simetryn	8.5	3.98E-02	EC50 and LC50	31	plants	Nagai, 2016
	terbuthylazine	7.2	3.13E-02	EC50	15	plants	Lewis and Thursby, 2018
	terbutryn	3.33	1.38E-02	EC50	11	algae	Larras et al.; 2012
ureas	daimuron	1000	3.73E+00	EC50 and LC50	7	plants	Nagai, 2016
	isoproturon	13.9	6.74E-02	EC50	11	algae	Larras et al.; 2012
	linuron	3.4	1.36E-02	EC50	10	plants	Lewis and Thursby, 2018
	diuron	1.9	8.15E-03	EC50	46	algae	Larras et al.; 2012
	cumyluron	910	3.01E+00	EC50 and LC50	6	plants	Nagai, 2016
miscellaneous	2,4-D	71	3.21E-01	EC50	6	plants	van den Brink, et al., 2006
	benfuresate	5500	2.15E+01	EC50 and LC50	6	plants	Nagai, 2016
	bentazone	4600	1.91E+01	EC50 and LC50	6	plants	Nagai, 2016
	benzofenap	16	3.71E-02	EC50 and LC50	6	plants	Nagai, 2016

	butachlor	2.8	8.98E-03	EC50 and LC50	6	plants	Nagai, 2016
	cafenstrole	7	2.00E-02	EC50 and LC50	10	plants	Nagai, 2016
	carfentrazone-C_2H_5	0.68	1.65E-03	EC50 and LC50	9	plants	Nagai, 2016
	diquat	0.6	3.26E-03	EC50	13	plants	Lewis and Thursby, 2018
	esprocarb	200	7.54E-01	EC50 and LC50	9	plants	Nagai, 2016
	fentrazamide	6.5	1.86E-02	EC50 and LC50	6	plants	Nagai, 2016
	indanofan	2	5.87E-03	EC50 and LC50	6	plants	Nagai, 2016
	mefenacet	71	2.38E-01	EC50 and LC50	9	plants	Nagai, 2016
	metolachlor	30.8	1.09E-01	EC50	28	plants	Lewis and Thursby, 2018
	molinate	530	2.83E+00	EC50 and LC50	8	plants	Nagai, 2016
	oxadiargyl	0.13	3.81E-04	EC50 and LC50	8	plants	Nagai, 2016
	pendimethalin	2	7.11E-03	EC50	6	plants	van den Brink, et al., 2006
	pentoxazone	0.067	1.89E-04	EC50 and LC50	6	plants	Nagai, 2016
	pretilachlor	2.5	8.02E-03	EC50 and LC50	9	plants	Nagai, 2016
	pyraclonil	0.56	1.78E-03	EC50 and LC50	6	plants	Nagai, 2016
	pyriminobac-CH_3	38000	1.05E+02	EC50 and LC50	6	plants	Nagai, 2016
	pyrimisulfan	3.4	8.11E-03	EC50 and LC50	7	plants	Nagai, 2016
	pyrazoxyfen	76	1.88E-01	EC50 and LC50	6	plants	Nagai, 2016
	quinoclamine	13	6.26E-02	EC50 and LC50	7	plants	Nagai, 2016
	tefuryltrione	3000	6.77E+00	EC50 and LC50	6	plants	Nagai, 2016
	thiobencarb	19	7.37E-02	EC50 and LC50	9	plants	Nagai, 2016

Table S7- Fungicides: selected HC_5 values (expressed both as μg L^{-1} and μmol L^{-1}), number and typologies of tested species to derive the SSD curves

Chemical class	Substance	HC_5 (μg L^{-1})	HC_5 (μmol L^{-1})	Endpoint(s)	N° endpoints (SSD)	Tested organisms	References
azoles	cyproconazole	171	5.86E-01	EC50	9	AQCOM	Maltby et al., 2009
	epoxiconazole	14	4.24E-02	EC50	6	AQCOM	Maltby et al., 2009
	fenbuconazole	244	7.24E-01	EC50	8	AQCOM	Maltby et al., 2009
	hydroxyisoxazole	29000	2.93E+02	EC50 and LC50	13	AQCOM	Nagai, 2016
	myclobutanil	220	7.62E-01	EC50	8	AQCOM	Maltby et al., 2009
	propiconazole	827	2.42E+00	EC50	19	vertebrates	Maltby et al., 2009
	tebuconazole	238	7.73E-01	EC50	9	AQCOM	Maltby et al., 2009
	tricyclazole	4000	2.11E+01	EC50 and LC50	17	AQCOM	Nagai, 2016
carbamates	benomyl	22	7.58E-02	EC50	19	AQCOM	Maltby et al., 2009
	carbendazim	8	4.18E-02	EC50	16	AQCOM	Maltby et al., 2009
	mancozeb	89	1.64E-01	EC50	18	AQCOM	Maltby et al., 2009
	maneb	48	1.81E-01	EC50	22	AQCOM	Maltby et al., 2009
	metiram (Zineb)	40	1.45E-01	EC50	23	AQCOM	Maltby et al., 2009
	thiram	1	4.16E-03	EC50	24	AQCOM	Maltby et al., 2009
	ziram	3	9.81E-03	EC50	12	vertebrates	Maltby et al., 2009
strobilurines	azoxystrobin	42	1.04E-01	EC50	17	AQCOM	Maltby et al., 2009
	fluoxastrobin	44	9.59E-02	EC50	14	AQCOM	Maltby et al., 2009
	kresoxim-CH_3	12	3.83E-02	EC50	15	AQCOM	Maltby et al., 2009
	picoxystrobin	17	4.63E-02	EC50	19	AQCOM	Maltby et al., 2009
	trifloxystrobin	8	1.96E-02	EC50	23	AQCOM	Maltby et al., 2009
miscellaneous	anilazine	115	4.17E-01	EC50	12	AQCOM	Maltby et al., 2009
	captafol	9	2.58E-02	EC50	13	AQCOM	Maltby et al., 2009
	captan	30	9.98E-02	EC50	24	fish	Maltby et al., 2009
	chlorothalonil	6	2.26E-02	EC50	46	AQCOM	Maltby et al., 2009
	cyprodinil	31	1.38E-01	EC50	18	invertebrates	Maltby et al., 2009
	dithianon	14	4.72E-02	EC50	11	AQCOM	Maltby et al., 2009
	dodine	58	2.02E-01	EC50	13	AQCOM	Maltby et al., 2009
	fluazinam	8	1.72E-02	EC50	27	AQCOM	Maltby et al., 2009
	fludioxonil	63	2.54E-01	EC50	8	AQCOM	Maltby et al., 2009
	flutolanil	866	2.68E+00	EC50	10	AQCOM	Maltby et al., 2009
	folpet	33	1.11E-01	EC50	21	invertebrates	Maltby et al., 2009
	hexachlorobutadiene	64	2.45E-01	EC50	12	AQCOM	Maltby et al., 2009
	HCB	11.54	4.05E-02	LC50	12	AQCOM	Wang et al., 2008
	iprobenfos	1100	3.81E+00	EC50 and LC50	22	AQCOM	Nagai, 2016
	isoprothiolane	5000	1.72E+01	EC50 and LC50	16	AQCOM	Nagai, 2016
	phthalide	7200	5.37E+01	EC50 and LC50	17	AQCOM	Nagai, 2016
	probenazole	1600	7.17E+00	EC50 and LC50	13	AQCOM	Nagai, 2016

	pyrazophos	63	1.69E-01	EC50	14	AQCOM	Maltby et al., 2009
	pyroquilon	17000	9.81E+01	EC50 and LC50	15	AQCOM	Nagai, 2016
	tolylfluanid	12	3.46E-02	EC50	17	AQCOM	Maltby et al., 2009

REFERENCES

EFSA (European Food and Safety Agency), 2007. Chlordane as undesirable substance in animal feed - Scientific Panel on Contaminants in the Food Chain. The EFSA Journal, 582, 1-53. https://efsa.onlinelibrary.wiley.com/doi/pdf/10.2903/j.efsa.2007.582.

Ellgehausen, H., D'Hondt, C., Fuerer, R., 1981. Reversed-phase chromatography as a general method for determining octan-1-ol/water partition coefficients. Pesticide Science, 12, 219-227. https://doi.org/10.1002/ps.2780120216.

Finizio, A., Vighi, M., Sandroni, D., 1997. Determination of n-octanol/water partition coefficient (Kow) of pesticides: critical review and comparison among methods. Chemosphere, 34, (1), 131-161.

Fisk, A.T., Rosenberg, B., Cymbalisty, C.D., Stern, G.A, Muir, D.C.G., 1999. Octanol/water partition coefficients of toxaphene congeners determined by the "slow-stirring" method. Chemosphere, 39, 2549-2562. https://doi.org/10.1016/S0045-6535(99)00157-5.

Gandy, M., Corral, M., Mylne, J., Stubbs, K., 2015. An interactive database to explore herbicide physicochemical properties. Organic & Biomolecular Chemistry, 13, 5586-5590. https://doi.org/10.1039/C5OB00469A.

Giddings, J.M., Williams, W.M., Solomon, K.R., Giesy, J.P., 2014. Risks to aquatic organisms from use of chlorpyrifos in the United States. Rev Environ Contam Toxicol., 231, 119-62. doi: 10.1007/978-3-319-03865-0_5.

Han, S., Qiao, J., Zhang, Y., Yang, L., Lian, H., Xin, G., Chen, H., 2011. Determination of n-octanol/water partition coefficient for DDT-related compounds by RP-HPLC with a novel dual-point retention time correction. Chemosphere, 83, 131-136. https://doi.org/10.1016/j.chemosphere.2011.01.013.

Hansch, C., Leo, A., Hoekman, D., 1995. Exploring QSAR – hydrophobic, electronic, and steric constants. Washington, DC: American Chemical Society, p.101.

Larras, F., Bouchez, A., Rimet, F., Montuelle, B., 2012. Using bioassays and species sensitivity distributions to assess herbicide toxicity towards benthic diatoms. PLoS ONE, 7(8), e44458. https://doi.org/10.1371/journal.pone.0044458.

Leo, A.J., 1998. MedChem Database. Daylight Chemical Information Systems Inc.; Irvine, CA.

Lewis, M., Thursby, G., 2018. Aquatic plants: test species sensitivity and minimum data requirement evaluations for chemical risk assessments and aquatic life criteria development for the USA. Environmental Pollution, 238, 270-280. https://doi.org/10.1016/j.envpol.2018.03.003.

Maltby, L., Blake, N., Brock, T.C.M., van den Brink, P.J., 2005. Insecticide species sensitivity distributions: importance of test species selection and relevance to aquatic ecosystems. Environmental Toxicology and Chemistry, 24(2), 379-88. https://doi.org/10.1897/04-025R.1.

Maltby, L., Brock, T.C.M., van den Brink, P.J., 2009. Fungicide risk assessment for aquatic ecosystems: importance of interspecific variation, toxic mode of action, and exposure regime. Environtal Science & Technology 43(19), 7556-7563. doi: 10.1021/es901461c.

Matsushita, T., Morimoto, A., Kuriyama, T., Matsumoto, E., Matsui, Y., Shirasaki, N., Kondo, T., Takanashi, H., Kameya, T., 2018. Removals of pesticides and pesticide transformation products

during drinking water treatment processes and their impact on mutagen formation potential after chlorination. Water Research, 138, 67-76. https://doi.org/10.1016/j.watres.2018.01.028.

Mnif, W., Hassine, A.I.H., Bouaziz, A., Bartegi, A., Thomas, O., Roig, B., 2011. Effect of endocrine disruptor pesticides: a review. International Journal of Environmental Research and Public Health 2011, 8, 2265-2303. https://doi.org/10.3390/ijerph8062265.

Nagai, T., 2016. Ecological effect assessment by species sensitivity distribution for 68 pesticides used in japanese paddy fields. Journal of Pesticide Science, 41(1), 6-14. https://doi.org/10.1584/jpestics.D15-056.

O'Neil, M.J., 2013. The Merck index: an encyclopaedia of chemicals, drugs, and biologicals. Cambridge, UK: Royal Society of Chemistry. ISBN 9781849736701.

PPDB (Pesticides Property Database). available at http://sitem.herts.ac.uk/aeru/ppdb/en/index.htm

Rico, A., Waichman, A.V., Geber-Corrêa, R. van den Brink, P.J., 2011. Effects of malathion and carbendazim on amazonian freshwater organisms: comparison of tropical and temperate species sensitivity distributions. Ecotoxicology, 20, 625–634. https://doi.org/10.1007/s10646-011-0601-9.

Roberts, T., Hutson, D., 1999. Metabolic pathways of agrochemicals: part 2: insecticides and fungicides. Royal Society of Chemistry, Cambridge. https://doi.org/10.1039/9781847551375

UCDAVIS (University of California), 2010a. Water quality criteria report for bifenthrin - phase III: application of the pesticide water quality criteria methodology. (available at: https://www.waterboards.ca.gov/rwqcb5/water_issues/tmdl/central_valley_projects/central_valley_pesticides/criteria_method/bifenthrin/final_bifenthrin_criteria_rpt.pdf).

UCDAVIS (University of California), 2010b. Water quality criteria report for cyfluthrin - phase III: application of the pesticide water quality criteria methodology. (available at: https://www.waterboards.ca.gov/rwqcb5/water_issues/tmdl/central_valley_projects/central_valley_pesticides/criteria_method/2015_reports/2015_cyfluthrin_rpt.pdf).

UNEP (United Nations Environment Programme), 2007. Report of the Persistent Organic Pollutants Review Committee on the work of its third meeting. Revised risk profile on chlordecone. UNEP/POPS/POPRC.3/20/Add.10. http://www.pops.int/documents/meetings/poprc/POPRC3/POPRC3_Report_e/POPRC3_Report_add10_e.pdf

USEPA (United States Environmental Protection Agency) CompTox database. CompTox Chemicals Dashboard (available at https://comptox.epa.gov/dashboard/).

van den Brink, P.J., Blake, N., Brock, T.C.M., Maltby, L., 2006. Predictive value of species sensitivity distributions for effects of herbicides in freshwater ecosystems. Human and Ecological Risk Assessment: An International Journal, 12(4), 645-674. doi: 10.1080/10807030500430559.

van Wijngaarden, R.P.A., Barber, I., Brock, T.C.M., 2009. Effects of the pyrethroid insecticide gamma-cyhalothrin on aquatic invertebrates in laboratory and outdoor microcosm tests. Ecotoxicology 18, 211-224. https://doi.org/10.1007/s10646-008-0274-1.

Verschueren, K., 2001. Handbook of Environmental Data on Organic Chemicals, 4th Edition. New York, NY,, Van Nostrand Reinhold Co.

Wang, B., Yu, G., Huang, J., Hu, H., 2008. Development of species sensitivity distributions and estimation of HC5 of organochlorine pesticides with five statistical approaches. Ecotoxicology, 17, 716-724. https://doi.org/10.1007/s10646-008-0220-2.

WHO/FAO (World Heath Organization/Food and Agriculture Organization), 2004. Pesticides residues in food – 2003: evaluations, parte 1. FAO plant and protection paper 177. Joint meeting of the FAO panel of experts on pesticide residues in food and the environment and the WHO core assessment group. Geneva, Switzerland, 15-24 September 2003.
